# A comparative evaluation of concordance and speed between smartphone app-based and artificial intelligence web-based cephalometric tracing software with the manual tracing method: A cross-sectional study

**DOI:** 10.4317/jced.60899

**Published:** 2024-01-01

**Authors:** Shantam Gupta, Shravan Shetty, Srikant Natarajan, Supriya Nambiar, Ashith MV, Saloni Agarwal

**Affiliations:** 1 Department of Orthodontics and Dentofacial Orthopaedics, Manipal College of Dental Sciences Mangalore, Manipal Academy of Higher Education, Manipal, Karnataka, India; 2 Department of Oral Pathology and Microbiology, Manipal College of Dental Sciences Mangalore, Manipal Academy of Higher Education, Manipal, Karnataka, India

## Abstract

**Background:**

This study compared the accuracy and speed of cephalometric analysis using an artificial intelligence web-based method and a smartphone app-based system with manual cephalometric analysis as the reference standard.

**Material and Methods:**

In this cross-sectional study, the lateral cephalograms were analysed using four methods: manual tracing, smartphone app tracing, artificial intelligence web-based automated tracing without manual landmark identification correction and artificial intelligence web-based automated tracing with manual landmark identification correction. The principal investigator obtained linear and angular cephalometric measurements to compare the accuracies of the four methods being assessed. Additionally, the duration required for landmark identification and subsequent analysis was recorded.

**Results:**

The analyses included 40 lateral cephalograms that were selected based on the inclusion and exclusion criteria. Very good to excellent agreement was observed in the accuracies of the artificial intelligence web-based and smartphone app-based systems compared with manual tracing (interclass correlation coefficient values ranging from 0.707 to 0.9, *p*< 0.001). Of the artificial intelligence web-based systems, the method without correction of automated landmark detection showed less reliable measurements than the other methods. Cephalometric analysis using artificial intelligence web-based and smartphone app-based systems consumed less time than manual tracing (*p*< 0.001).

**Conclusions:**

Artificial intelligence web-based automated tracing with manual landmark identification correction and smartphone-based app provide results that are comparable to those from the manual tracing method. However, artificial intelligence web-based systems require improvements in terms of automated landmark identification to obtain results that are similar to those from the other methods being assessed.

** Key words:**Artificial Intelligence, Cephalometry, Computer software, Mobile application.

## Introduction

Broadbent and Hofrath introduced cephalometrics in 1931; since then, it has become an integral part of the analysis of malocclusion and one of the standardised diagnostic methods in orthodontic research and practice. Conventional manual cephalometrics is performed by pencil-tracing radiographical points on acetate overlays and measuring the readings using a protractor. Despite its extensive use in orthodontics, the method has certain drawbacks, including the amount of time consumed to perform each analysis. Moreover, inaccuracy while hand tracing, identifying landmarks and measuring is a major concern (1).

Cephalometric tracing can be performed using digitisers as well as directly on a screen that displays digital pictures, thanks to technological advancements in computer science. More than 350 orthodontic applications are currently available, many of which are open source (2). When it comes to orthodontic applications designed to perform cephalometric analysis, contradictory findings exist regarding the reliability of cephalometric analysis app compared with manual tracing software (3). The common feature of all these digital cephalometric tracing platforms, whether used on a smartphone, computer, or Tablet, is that the orthodontist has to individually mark the anatomic points during tracing, which makes the cephalometric program only semiautomated (4,5). Various software programs, such as YOLOv3 (You only look once - version 3) created by Redmond and Single Shot Multibox Detector by Liu (6-8), are available on the market, which aid in automated landmark identification. Comparative studies are necessary owing to the proliferation of smartphone applications, the availability of computer-aided cephalometric tracing programs, and the imprecision of commercially available software. Recent studies have reported that fully automatic cephalometric software powered by artificial intelligence cannot locate landmarks on the lateral cephalometric radiograph with complete accuracy. The software requires manual intervention from the observer to reduce the margin of error (9,10). Therefore, this study evaluated artificial intelligence web-based automated tracing with and without manual landmark identification correction so that the difference in accuracy and the time spent in landmark identification correction could be assessed after manual intervention from the investigator. This assessment will allow clinicians and orthodontists to make a knowledge-based choice regarding the best method and analysis techniques (11). Regardless of the use of fully automated, semiautomated, or manual landmark identification and analysis, any system should be reliable, precise, and most importantly, highly reproducible.

Therefore, this study aimed to compare the accuracy and time required to perform cephalometric analysis using an artificial intelligence system web-based method with and without landmark identification correction and a smartphone app-based system with manual cephalometric analysis as the reference standard. The null hypothesis was that there are no significant differences among the cephalometric analysis methods in terms of their accuracy or tracing time.

## Material and Methods

Institutional ethical clearance was obtained before commencing the study (protocol ref no: 20070). This cross-sectional study was performed for a period of 6 months. In this study, lateral cephalograms of patients who had visited the Department of Orthodontics and Dentofacial Orthopaedics from January 2022 to June 2022 were collected. Radiographs were screened based on selection criteria. Lateral cephalograms of patients aged 18-40 years were included. The inclusion criteria were having a cephalometric radiograph acquired by the same film-based cephalometric machine, having all of the landmarks to be examined within the image’s boundaries and being able to be identified, and having all radiographs acquired in centric occlusion with the patients’ heads in their natural positions and lips relaxed. The exclusion criteria were unerupted/missing incisors, unerupted teeth overlying the incisor apices or artifacts hindering landmark identification, poor quality films, periapical pathology, craniofacial anomalies, any malposition of the head with the cephalometer, and ongoing fixed orthodontic treatment.

-Image acquisition

The lateral cephalograms were acquired with Planmeca ProMax S2-2D (Helsinki, Finland, 2008) using standard imaging techniques. The exposure parameters were 60–84 kV and 5–16 mA and an average exposure time of 18.7 s. Image analysis was performed using four methods: a. manual tracing; b. smartphone-based OneCeph app (version beta 9, Google Play Store, Google Inc, Mountain View, Calif); c. web-based fully automated tracing WEBCEPH™ software (AssembleCircle Corp., Seoul, Korea; version 1.0) with automated landmark detection; and d. web-based fully automated tracing WEBCEPH™ with manual correction of landmarks.

-Image analysis

Group A: Manual tracing: The films were 2.232 × 2.304 pixels, 150 dpi and 8 bits; a 0.5-mm lead pencil was used for manual tracings. All soft and hard tissue landmarks were traced. A ruler and a protractor were used to measure the angular and linear parameters.

**Figure 1:**
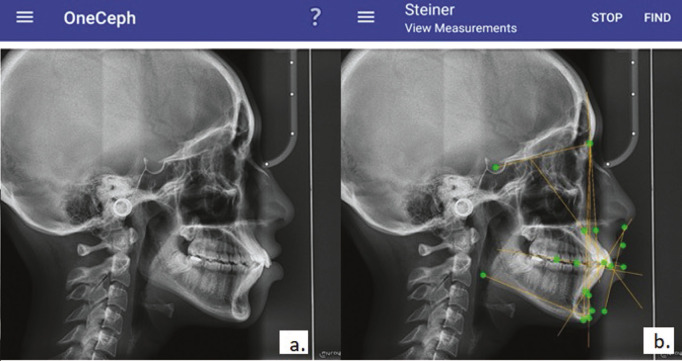
(a) Interface of smartphone app-based OneCeph app, (b) Analysis performed in OneCeph app.

Group B: Smartphone app-aided tracing: In the app-based tracing method, the OneCeph app was used. Using a standard computer, the digitised radiographs were uploaded as .jpeg files to the Android phone [OnePlus 9R, OnePlus Technology (Shenzhen) Co., Ltd.]. Calibration was performed by marking the known distance on the digitised cephalograms, following which cephalometric analysis was performed (Fig. 1a,b).

**Figure 2:**
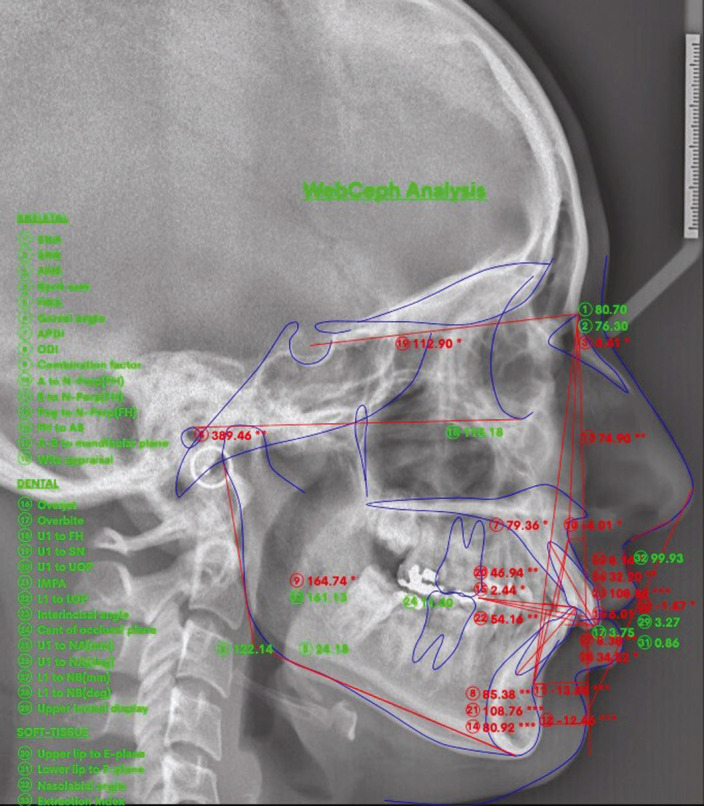
Analysis performed in Artificial Intelligence web-based WEBCEPH™ software.

Group C and D: Artificial intelligence web-based fully automated tracing: An online platform-based digital cephalometric software called WEBCEPH™ was used (Fig. 2). After entering the address www.webceph.com using a standard web browser (Google Chrome 64 bit), a new patient identification was created, and a “jpeg” formatted cephalometric X-ray image of the patient was uploaded. Subsequently, the artificial intelligence web-based WEBCEPH™ was utilized to calibrate the cephalogram using the calibration option in the software. The landmarks were identified by the software without any manual correction, and the analysis was performed based on these landmarks in group C. On the contrary, in group D, the landmarks were manually corrected after being identified by the software before performing the analysis.

-Time analysis

The speed was determined using a timer to calculate the time in seconds to identify landmarks and calculate the linear and angular measurements. The beginning and ending points for the manual cephalometric measurements were obtained by plotting the landmarks and measuring the angles and distances. Two timings were recorded for the web-based fully automated tracing. The first time recorded was when the system automatically recognised the anatomical locations and generated the analysis. The automatically recognised landmarks were manually revised, and the overall analysis time, i.e., the second timing for the web-based fully automated tracing, was recorded. The time analysis for smartphone app-aided tracing included plotting the landmarks used by the operator until measurements were generated.

-Statistical analysis 

**Figure 3:**
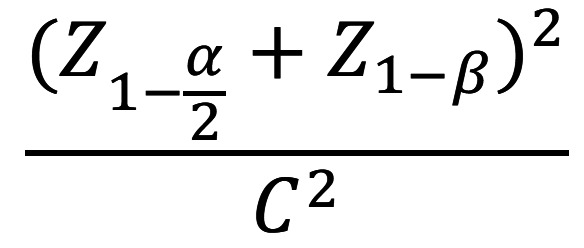
Formula.

A pilot study was conducted using 20 lateral cephalogram samples to measure the angular values of Sella–Nasion–Point A (SNA) and Sella–Nasion–Point B (SNB) cephalometric parameters. The Pearson’s R correlation coefficient between the artificial intelligence method and the manual tracing method was noted to be 0.720. The formula (Fig. 3):

where C was calculated as C = 0.5*ln[(1+|r|)/(1-|r|)], was used. The r value of 0.720 obtained from the pilot study and the Z values for an alpha error of 1% and a power of 99.9% were inputed, corresponding to the Z alpha and Z beta constants taken as 2.58 and 3.09, respectively, and the required sample size was determined to be 39.

The data obtained from this study were recorded on a Microsoft Excel™ 2019 spreadsheet, and statistical analyses were performed using the Statistical Package for Social Sciences, version 20.0, software (IBM Corp; Armonk, NY, USA). The agreement of the continuous variables for both angular and linear cephalometric measurements was estimated using the interclass correlation coefficient (ICC) for all four groups. The association of the mean values of the measurements was compared pairwise (constituting six pairs for four separate measurements) using the paired t-test. A *p* value of <0.05 was considered significant.

## Results

The analyses included 40 lateral cephalograms that were selected based on the inclusion and exclusion criteria. Testing the agreement between the angular measurements (SNA and SNB) showed very good agreement, with an ICC value of 0.707. The other variables [point A–Nasion–Point B (ANB), Sella–Nasion to Gonion–Gnathion (SN–GoGn), Upper Incisor to Nasion Point A (UI–NA) (degree), Upper Incisor to Nasion Point A (UI–NA) (mm), Lower Incisor to Nasion Point B (LI-NB) (degree), Lower Incisor to Nasion Point B (LI–NB) (mm), Occlusal–Sella Nasion (Occ–SN), Upper Lip–Aesthetic line (UL–Eline) and Lower Lip–Aesthetic line (LL–Eline)] showed ICC values of >0.9, which indicated excellent agreement (Table 1). Testing the association of the mean values among the four groups showed that the significant differences in measurements were because of the WEBCEPH without manual landmark identification correction, wherein the values were overestimated (mean values were more than the actual values) in terms of SNA, SNB, ANB and LL-Eline. However, SN–GoGn, UI to NA (degree), UI to NA (mm) and Occ–SN were underestimated (Table 2). Therefore, of the four groups being assessed, the artificial intelligence web-based method without correction of automated landmark detection showed the least reliable measurements. Cephalometric analysis using artificial intelligence web-based and smartphone app-based systems consumed less time than manual tracing (*p* < 0.001) (Table 2).

**Table 1:**
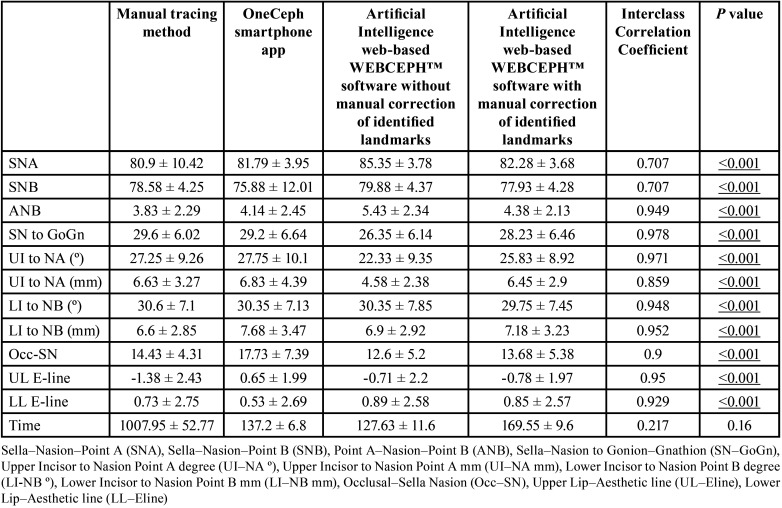
Interclass correlation coefficient used to compare the intra-observer variability / agreement between the parameters.

**Table 2:**
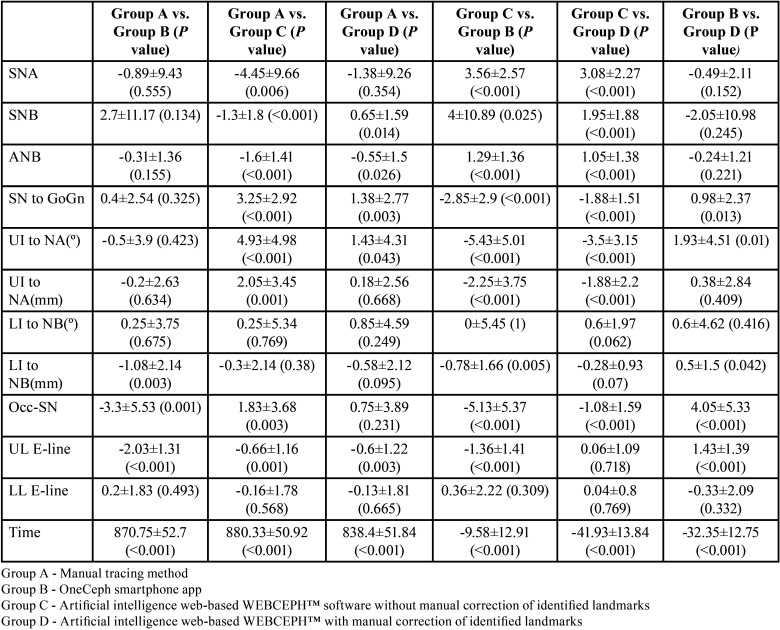
Comparison of the differences between the individual methods (Paired t test).

## Discussion

Digitised systems are being increasingly used in cephalometry owing to rapidly evolving technological advances. Cephalometric analysis can be easily accessed via web-based and smartphone applications, which allows automated cephalogram tracing. Regardless of which method is being utilised, excellent accuracy is needed for cephalometric tracing. Therefore, this study focused on comparing the accuracy of the smartphone-based OneCeph app and the artificial intelligence web-based fully automated tracing with the WEBCEPH™ software utilizing automated landmark detection and manual landmark correction features with the manual tracing method. The key finding of this study was that automated tracing with artificial intelligence web-based WEBCEPH™ is significantly faster than the other techniques employed, and there is scope for improvement for the software to become more reliable in terms of the accuracy of specific landmarks. This finding agrees with a previous study that used artificial intelligence (14). After manual correction of landmarks on the WEBCEPH™ software, the measurements obtained were more accurate than those from merely using the automated landmark identification feature of artificial intelligence web-based WEBCEPH™. The use of software-based cephalometrics may reduce the errors that might occur during manual tracing with a protractor and ruler (15,16). A few measurements, especially those involving mandibular and maxillary incisors, are challenging. These structures have been shown to exhibit low reliability not only in manual but also in digital tracings despite possessing the features of zooming and filtering (17). Furthermore, previous investigations have reported that gonion and gnathion are sources of error and are inconsistent in nature. However, these results are not in accordance with the findings of this study, i.e., the measurements revealed no significant differences and exhibited a good correlation, with the most reliable system being the smartphone-based OneCeph application. With the use of WEBCEPH™ without manual correction, the linear values were less reliable than angular values. These results agree with the observations of other investigations (18,19).

Correction of automated landmark identification was performed for WEBCEPH™ for the manual correction group, which yielded a significantly better correlation with the manual tracing method than mere automated identification with the artificial intelligence web-based WEBCEPH™ group. In addition, the resolution of the images used is an essential factor that determines the validity of the results. Digital images of 8 bits and 150 dpi are sufficient for clinical purposes (20). This study used a resolution of 150 dpi for all four groups to allow easy comparison and standardization.

This study found that the digital measurements consumed far less time than the manual tracing approach, which is consistent with previous findings (21,22). The analysis performed with the smartphone-based OneCeph and the artificial intelligence web-based WEBCEPH™ was nine times faster than that performed with the manual tracing method. The time taken to conduct a cephalometric analysis should not include the time required to provide a diagnosis and treatment plan. Although the shortest time was obtained with automated landmark identification using artificial intelligence web-based WEBCEPH™, its measurements were less reliable than those from the other methods. Therefore, the validity and reliability of landmark identification and tracing are of paramount importance and are superior to the time consumed. Nevertheless, it should be noted that manual correction of artificial intelligence web-based WEBCEPH™ and smartphone-based OneCeph landmarks yielded similar measurements compared with the manual tracing method. Moreover, the time consumed for the analysis was considerably less, which makes these methods promising for use in clinical orthodontic practice.

## Conclusions

Based on the results of the study, it could be stated that the artificial intelligence web-based automated tracing with manual landmark identification correction and the smartphone-based app provide results that are comparable to those from the manual tracing method. However, artificial intelligence web-based systems require improvements in terms of automated landmark identification to yield results similar to those from the other methods being assessed. Cephalometric analysis using the artificial intelligence web-based system and the smartphone app-based system consumed less time than manual tracing.

## References

[1] Albarakati SF, Kula KS, Ghoneima AA (2012). The reliability and reproducibility of cephalometric measurements: a comparison of conventional and digital methods. Dentomaxillofac Radiol.

[2] Gupta G, Vaid NR (2017). The World of Orthodontic apps. APOS Trends Orthod.

[3] Livas C, Delli K, Spijkervet FKL, Vissink A, Dijkstra PU (2019). Concurrent validity and reliability of cephalometric analysis using smartphone apps and computer software. Angle Orthod.

[4] Celik E, Polat-Ozsoy O, Toygar Memikoglu TU (2009). Comparison of cephalometric measurements with digital versus conventional cephalometric analysis. Eur J Orthod.

[5] Mosleh MAA, Baba MS, Malek S, Almaktari RA (2016). Ceph-X: development and evaluation of 2D cephalometric system. BMC Bioinformatics.

[6] Redmon J, Farhadi A (2018). Yolov3: an incremental improvement. arXiv.

[7] Liu W, Anguelov D, Erhan D, Szegedy C, Reed SE, Fu CY (2016). SSD: Single Shot MultiBox Detector. European Conf Comput Vis. Springer International Publishing.

[8] Park JH, Hwang HW, Moon JH, Yu Y, Kim H, Her SB (2019). Automated identification of cephalometric landmarks: Part 1-Comparisons between the latest deep-learning methods YOLOV3 and SSD. Angle Orthod.

[9] Duran GS, Gökmen Ş, Topsakal KG, Görgülü S (2023). Evaluation of the accuracy of fully automatic cephalometric analysis software with artificial intelligence algorithm. Orthod Craniofac Res.

[10] El-Dawlatly M, Attia KH, Abdelghaffar AY, Mostafa YA, Abd El-Ghafour M (2023). Preciseness of artificial intelligence for lateral cephalometric measurements. J Orofac Orthop.

[11] Shettigar P, Shetty S, Naik RD, Basavaraddi SM, Patil AK (2019). A Comparative Evaluation of Reliability of an Android-based App and Computerized Cephalometric Tracing Program for Orthodontic Cephalometric Analysis. Biomed Pharmacol J.

[12] Schulze RKW, Gloede MB, Doll GM (2002). Landmark identification on direct digital versus film-based cephalometric radiographs: A human skull study. Am J Orthod Dentofac Orthop.

[13] Paixão MB, Sobral MC, Vogel CJ, de Araujo TM (2010). Comparative study between manual and digital cephalometric tracing using Dolphin Imaging software with lateral radiographs. Dental Press J Orthod.

[14] Moreno M, Gebeile-Chauty S (2022). Comparative study of two software for the detection of cephalometric landmarks by artificial intelligence. Orthod Fr.

[15] Tsolakis IA, Tsolakis AI, Elshebiny T, Matthaios S, Palomo JM (2022). Comparing a Fully Automated Cephalometric Tracing Method to a Manual Tracing Method for Orthodontic Diagnosis. J Clin Med.

[16] Lagravère MO, Low C, Flores-Mir C, Chung R, Carey JP, Heo G (2010). Intraexaminer and interexaminer reliabilities of landmark identification on digitized lateral cephalograms and formatted 3-dimensional cone-beam computerized tomography images. Am J Orthod Dentofac Orthop.

[17] Baumrind S, Frantz RC (1971). The reliability of head film measurements. Am J Orthod.

[18] Trpkova B, Major P, Prasad N, Nebbe B (1997). Cephalometric landmarks identification and reproducibility: a meta analysis. Am J Orthod Dentofacial Orthop.

[19] Chan CK, Tng TH, Hägg U, Cooke MS (1994). Effects of cephalometric landmark validity on incisor angulation. Am J Orthod Dentofac Orthop.

[20] Leonardi R, Giordano D, Maiorana F, Spampinato C (2008). Automatic cephalometric analysis: A systematic review. Angle Orthod.

[21] Prince STT, Srinivasan D, Duraisamy S, Kannan R, Rajaram K (2023). Reproducibility of linear and angular cephalometric measurements obtained by an artificial-intelligence assisted software (WebCeph) in comparison with digital software (AutoCEPH) and manual tracing method. Dental Press J Orthod.

[22] Gupta SP, Dahal S, Rauniyar S (2021). Reproducibility and speed of cephalometric tracing between manual versus digital method. Orthodontic Journal of Nepal.

